# Primary appendiceal mucinous adenocarcinoma mimicking bladder carcinoma: A case report and review of the literature

**DOI:** 10.3892/ol.2014.1842

**Published:** 2014-01-30

**Authors:** KAI DENG, CHENG-QI ZHANG, GUANG-LI WANG, WEI LI

**Affiliations:** Department of Radiology, Shandong Provincial Qianfoshan Hospital Affiliated to Shandong University, Jinan, Shandong 250014, P.R. China

**Keywords:** appendix, mucinous adenocarcinoma, invade, bladder carcinoma

## Abstract

Primary appendiceal mucinous adenocarcinoma is a rare entity, while appendiceal carcinoma invading the urinary bladder is even rarer. The current study presents the case of a 54-year-old male with primary appendiceal mucinous adenocarcinoma, without gastrointestinal symptoms, presenting only with signs of bladder irritation. Abdominal computed tomography scans demonstrated a mass at the right anterior level of the bladder. During intraoperative exploration, the mass was detected to connect and adhere to the ileocecal junction, and normal appendix tissues were not found. The frozen section revealed a mucinous adenocarcinoma of the appendix and the patient immediately underwent a subsequent hemicolectomy. The present case indicates that a diagnosis of a primary appendiceal mucinous carcinoma must considered by radiologists and clinicians for patients who do not exhibit gastrointestinal symptoms, but show involvement of the nearest organs and the bladder wall.

## Introduction

Primary adenocarcinoma of the appendix is a rare malignancy with ~0.12 cases per 1,000,000 individuals diagnosed annually (1). The condition was first described by Berger in 1882 (2). Primary epithelial malignancies of the appendix are divided into three categories, including mucinous adenocarcinomas, intestinal-type adenocarcinomas and signet ring cell carcinomas (3–5). The mucinous adenocarcinoma is the most frequent histological type among the identified appendiceal carcinomas (1). As an uncommon clinical entity, a primary adenocarcinoma of the appendix only amounts for 0.01–0.2% of all gastrointestinal neoplasms (6) and it is extremely difficult to diagnose prior to surgical inspection; ≤50% of cases are diagnosed during intraoperative exploration of the peritoneal cavity (7–9). The diagnosis of primary appendiceal mucinous adenocarcinoma usually depends on the pathology following appendectomy or other explorative surgical procedures (10). The current case report presents a 54-year-old male with primary appendiceal mucinous adenocarcinoma mimicking urinary bladder carcinoma.

## Case report

**Figure 1:**
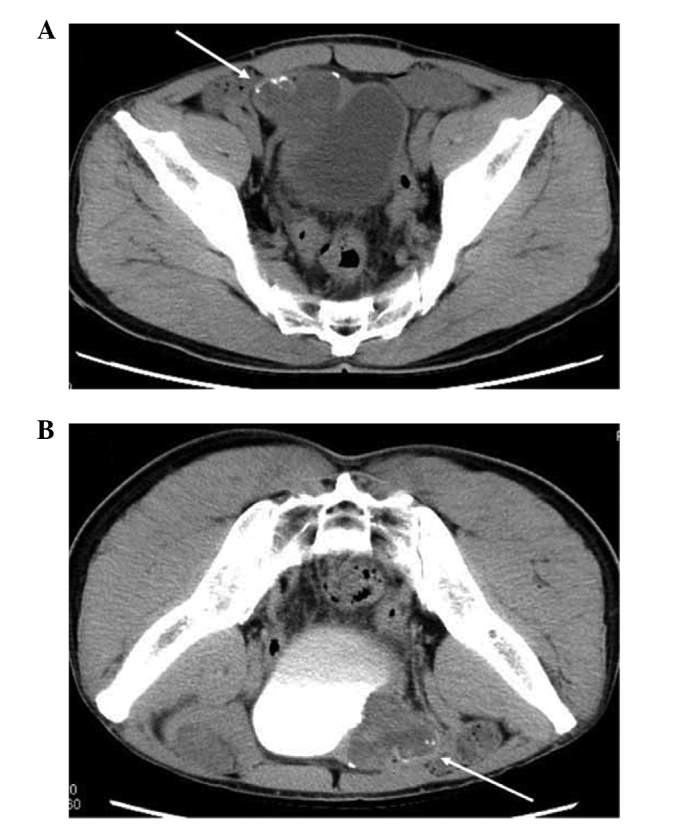
Enhanced abdominal computed tomography scans (transverse images). (A) Plain scan demonstrating a hypodense mass at the level of the right anterior bladder wall with sporadic calcifications in the peripheral area, as indicated by the arrow. (B) In postcontrast imaging (prone position), the mass was moderately and heterogeneously enhanced, as indicated by the arrow.

**Figure 2:**
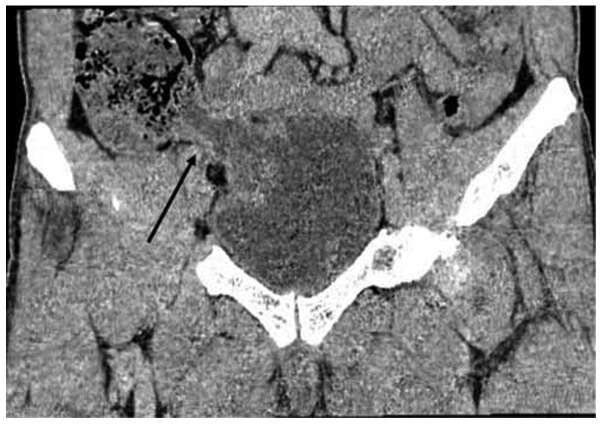
Coronal multiplanar reconstruction (MPR) of the computed tomography image showing that the mass was connected to the ileocecal junction along the appendix and that the density of the liquid content was low, as indicated by the arrow.

A 54-year-old previously healthy male presented at the Shandong Provincial Qianfoshan Hospital Affiliated to Shandong University (Jinan, China) with the clinical presentation of continuous frequent micturition, urgent urination and odynuria for approximately six months. The patient did not have a fever or any gastrointestinal symptoms. A physical examination was normal and laboratory tests showed microscopic, but no gross, hematuria. The level of tumor markers, carcinoembryonic antigen and carbohydrate antigen 19-9, were normal. A previous ultrasound at the patient’s local hospital showed a bladder tumor (only described orally by the patient without providing the formal diagnostic report). The enhanced abdomen computed tomography (CT) scan revealed a hypodense mass (5.4×4.6 cm at the maximum section) at the level of the right anterior bladder wall, with sporadic calcifications in the peripheral area. The mass was moderately and heterogeneously enhanced in postcontrast images (Fig. 1). From the coronal multiplanar reconstruction (MPR) imaging, the mass was shown to connect with the ileocecal junction along the area of the appendix, and the density of the liquid content was low (Fig. 2). This sign indicated that the mass may be closely associated with the intestinal tract, although it appeared to be a urinary bladder carcinoma. The patient provided written informed consent.

During the surgery, a mass with a maximum diameter of 5 cm was identified at the level of the right anterior bladder wall, markedly adhesive to the surrounding tissues. Following dissection of the peritoneum, the mass was detected to connect and adhere to the ileocecal junction, and normal appendix tissues were not found. Following consultation, gastrointestinal surgery experts considered that the tumor was of appendiceal origin, invading the urinary bladder. The frozen section revealed a mucinous adenocarcinoma of the appendix, and the patient immediately underwent a subsequent hemicolectomy. The final histological examination confirmed the presence of a primary appendiceal mucinous adenocarcinoma infiltrating the appendix and urinary bladder wall. The pathological staging was as follows: TNM classification, pT4N0M0; and Dukes’, Astler-Coller classification, stage B.

## Discussion

Histologically, adenocarcinomas of the appendix are classified into three distinct types: Mucinous, intestinal and signet ring cell. Various controversies in the field of appendiceal mucinous neoplasms have arisen over the past several decades, focusing on the classification of appendiceal mucinous tumors and the nature, classification and clinical significance of pseudomyxoma peritonei (11). Invasive adenocarcinomas of the appendix are rare, accounting for only 4–6% of primary malignant appendiceal neoplasms, according to a study by Hananel *et al* (12). The pre-operative diagnosis of primary appendiceal carcinoma is invariably difficult since the clinical presentation is usually non-specific. Therefore, appendiceal carcinoma is always neglected or misdiagnosed. The majority of cases are diagnosed during intraoperative exploration (13,14). The identification of a previously reported series of appendiceal neoplasms is difficult due to their rarity and the increased rarity of appendiceal carcinoma cases also invading the urinary bladder. Taverna *et al* (9) described the features and rarity of appendiceal carcinoma invading the urinary bladder. The appendiceal carcinoma usually involved the posterior bladder wall initially and subsequently the anterior bladder lesion was a secondary localization due to the anatomical relationship between the appendix and the bladder.

In the present case, the patient did not exhibit any gastrointestinal symptoms, with signs of bladder irritation, existing since morbidity, the only clinical presentation. Ultrasound and abdomen enhanced CT scans revealed a urinary bladder mass. Combined with the laboratory test results and symptoms, an initial diagnosis of urinary bladder carcinoma was made. However, from the CT MPR images, the mass was found to connect with the ileocecal junction, and the density of the liquid content was low. The region of the lesion was in line with the appendix. Therefore, the primary diagnosis was changed. It was considered that the lesion had originated from the intestinal tract, then invaded the bladder, and that the appendix was the most likely organ of this origin. This was confirmed by explorative surgery, followed by a subsequent hemicolectomy due to the frozen section analysis.

In the present case, the patient’s clinical presentation was confusing and the rarity of an appendiceal carcinoma invading the urinary bladder led us to initially ignore the possibility of its presence. The signs and symptoms of an appendiceal adenocarcinoma were not specifically apparent, but the CT MPR technique was useful to identify a correlation with the appendix. Therefore, a diagnosis of a primary appendiceal mucinous carcinoma must be considered by radiologists and clinicians for patients who do not exhibit gastrointestinal symptoms, but show involvement of the nearest organs and the bladder wall. It is possible that CT MPR images may provide more information.
